# LOLS Research in Technology for the Development and Application of New Fiber-Based Sensors

**DOI:** 10.3390/s120302654

**Published:** 2012-02-28

**Authors:** João Coelho, Marta Nespereira, Catarina Silva, José Rebordão

**Affiliations:** Laboratory of Optics, Lasers and Systems, Department of Physics, Faculty of Sciences, University of Lisbon, Polo do Lumiar, Estrada do Paço do Lumiar, 22, Building D, 1649-038 Lisboa, Portugal; E-Mails: mcnespereira@fc.ul.pt (M.N.); catarina.silva@fc.ul.pt (C.S.); jmnrebordao@fc.ul.pt (J.R.)

**Keywords:** laser processing, fiber-based sensors, encapsulation, refractive-index modulation, laser micropatterning

## Abstract

This paper presents the research made at the Laboratory of Optics, Lasers and Systems (LOLS) of the Faculty of Sciences of University of Lisbon, Portugal, in the field of fiber-based sensors. Three areas are considered: sensor encapsulation for natural aqueous environments, refractive index modulation and laser micropatterning. We present the main conclusions on the issues and parameters to take in consideration for the encapsulation process and results of its design and application. Mid-infrared laser radiation was applied to produce long period fiber gratings and nanosecond pulses of near-infrared Q-switch laser were used for micropatterning.

## 1. Introduction

The Laboratory of Optics, Lasers and Systems (LOLS) of the Faculty of Sciences of the University of Lisbon has been working for more than 20 years in the field of optics and lasers, although in the previous context of the no-longer existing *Instituto Nacional de Engenharia, Tecnologia e Inovação*, INETI. LOLS has a long experience on the development of optical techniques and laser processing of materials with activities in support of Portuguese industry, namely in the fields of defense, space, astrophysics and industrial manufacturing. In recent years, in cooperation with the Institute for Systems and Computer Engineering of Porto (INESC-Porto) interesting synergies have been identified in the area of fiber sensor devices.

The starting point in this field was the study of the encapsulation issues regarding the application of fiber sensors in harsh aqueous environments. The identification of the main challenges allowed developing a prototype for application in a real environment and set the main parameters for the development of future devices. Regarding the development of fiber sensors, targeted for application in rivers or maritime environments (or others, like the temperature and salinity gradients in solar ponds), two main research fields are being pursued: the application of laser radiation in the writing of long period fiber gratings and micropatterning of fibers. The two main laser sources used so far were CO_2_ and Nd:YAG lasers.

Besides experimental testing, the group is working in modeling the physical processes involved in the laser-fibers interaction. This is been done mainly through finite element method (FEM) using COMSOL Multiphysics™ with the purpose of obtaining a model capable of simulate the overall physical processes. Although at this moment we have only developed a thermo-mechanical model, its future development will consider the contribution of the different strains acting on the fiber and the overall influence in the refractive index changes.

The following sections, will describe the work already done in the development and application of fiber-based sensors. A resume of the state of our research in each field is presented and future goals and current developments will be pointed out.

## 2. Encapsulation of Fiber Sensors for Aqueous Natural Environments

In the field of aqueous environment studies, fiber sensors (as those based in Long Period Fiber Gratings, or LPFG) can be very attractive for real time monitoring of several physical parameters, such as temperature and salinity. However, in real *in situ* conditions, where mechanical loads and contamination with algae and other organic materials must be taken into account, the removal of the fiber jacket makes these devices very fragile. This is particularly true in natural aqueous media where the existence of biological activity is added to other debris that can not only change the sensing capability but also severely damage the entire sensing head. Following a challenge regarding the installation of LPFG fiber sensors in the Ria de Aveiro, Portugal, a system was developed to operate at half depth, inserted in the optical cable, thus protecting the optical fiber [1].

**Figure 1.**
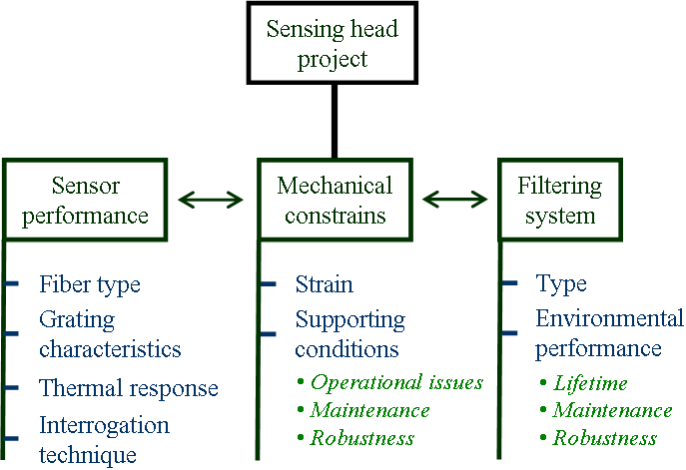
Schematic of the issues and parameters to take in consideration when projecting a sensing head to operate in natural aqueous environments.

When considering this application, the project of the sensing head must address a set of issues and parameters [2]. As schematized in Figure 1, we basically have to consider three main subjects: the sensor performance, which must be defined accordingly with the expected measurement needs, the mechanical constrains, that should take in consideration the encapsulation requirements, and the filtering system, which plays an important role for the *in situ* measurement capability during an acceptable period of time. Although the main focus was made on encapsulating LPFG sensors, most of these issues are common to other types of fibers-based sensors.

In this area, LOLS work was mainly based in the study and design of the sensing head based on the analysis of the mechanical constrains and on the requirements for the filtering system. Future developments in this field involve longer testing periods and an *in situ*, online, full set of measurements using a dedicated sensor head.

### 2.1. Mechanical Constrains

Capability to correctly support the fiber-based sensor is perhaps the major goal of the mechanical apparatus. One of the main constraints is that the LPFGs have intrinsic sensitivity to bending curvature, which consequently influences its sensitivity to the various parameters to be measured. Thus, in practical applications, it becomes necessary to design a support system that can avoid these cross sensitivities. A particular solution tested at LOLS was a spring-based system designed to apply a constant force to the grating regardless of the external handling conditions. The compression string must be carefully projected and the design should specify its length and number of active coils, as well as the wire’s diameter, based on the required compressive force, strings diameter and its material elastic properties.

As expected, being a device to operate in direct contact with the aqueous medium, the choice of the material to be used, not only in the spring but also in the overall supporting device, is of prime importance. Resistance to corrosion and structural rigidity are the main characteristics that must be taken in consideration, while machinability and price are also usually considered. Being intended to be a fully functional but pre-industrial prototype (and not supposed to operate for long periods of time), the device developed at LOLS was made of aluminum (1060 Alloy), while the spring was made of stainless steel. A cylindrical shape was chosen in order to allow the insertion of an outer mud filtrating membrane and a protective mechanical grid. The sensor element is surrounded by enough space to promote the flow of water allowing updating salinity measurements. The optical cable is fixed at both ends by mechanically blocking the fiber at the head’s support, leaving about 11 cm of exposed optical fiber. The coil spring was scaled by imposing a force (compression) of 20 g over a diameter of 40 mm. The spring, with a free length of 130 mm, was made of stainless steel with 14 active coils and a wire diameter of 1 mm.

**Figure 2.**
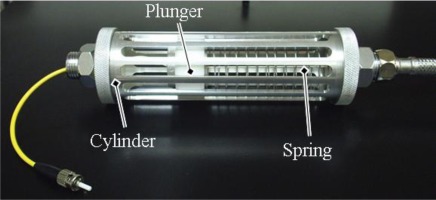
Picture of an encapsulation device designed for fiber-based sensors in aqueous environments.

The final aspect to be taken in consideration is that the device will have to be subjected to maintenance operations during its operational lifetime. This implies that the project should take this in consideration in order to guarantee that the replacement of filters, cleaning of the system and replacement of sensors are fast and easily accomplished. Figure 2 presents a picture of the device developed and implemented in the Ria de Aveiro, Portugal.

### 2.2. Filtering System

The system hosting the LPGs requires adequate filtration in order to prevent the deposition of organic materials and the accumulation of particles around the sensor element, which would disturb the measurement. The filtration process to protect the sensing head is essentially based on the passage of fluid through a porous or permeable material. The encapsulation of the sensor includes two levels of filtration: one to retain large particles and another to retain mud usually existing in natural aqueous environments. For filtrating large particles, it is common to use lower pore sizes in order to prevent the deposition of waste or algae. The filtration of mud is achieved through membrane separation and its efficiency depends on factors such as the properties of the membrane and fluid and operating conditions (pressure, temperature, turbulence).

Preliminary *in situ* tests allowed identifying the best filters. Three types of membranes were analyzed: two made of nylon (with different mesh sizes) and other of fiberglass. After one month submersed, the membranes were analyzed regarding their state of blockage, using an optical microscope [1,2]. Inside the vessel, another membrane was placed and a similar analysis was made in order to have an idea of the amount of impurities that have passed the outer filter. It was found that the fiber glass membrane was not adequate (it was completely destroyed by the environment) and that the nylon membrane with larger pore size (100 μm) was fully saturated. In opposition, the nylon membrane with half pore size still had open pores and was therefore considered the best solution for the filtrating system. Experience points out that, although capable of supporting a one month sensing campaign, a comfortable rule of thumb should be to replace the filtering system every two weeks.

## 3. Long Period Fiber Gratings Written by Laser

There are several techniques to write LPFGs in optical fibers, for example using ultraviolet (UV) light [3,4] or by electric discharges [5]. Mid-infrared radiation (MIR) has also been used to produce LPFGs by using CO_2_ laser technology [6,7]. After the first results in 1998 [7], it was realized that this fabrication process generates devices with excellent characteristics, particularly in what concerns their stability at very high temperatures (up to 1,000 °C). This technique to fabricate LPFGs has therefore became popular and R&D for its optimization has continuously been reported [8–10].

The standard LPFG fabrication processes allows writing single devices, *i.e.*, with a spectral response associated with coupling of the core mode to a specific cladding mode. There are situations where it is advantageous to fabricate LPFG structures with coupling from the core mode to several cladding modes, which should give a spectral response with characteristics adequate to advanced sensing. With the techniques indicated, it is feasible to fabricate these structures writing sequentially several gratings, each one associated with a specific cladding mode. However, the process is not efficient and a much better approach would be to perform the writing of the fiber in a single step using patterned light, with a spatial intensity distribution adequate to generate the material refractive index modulation associated with the required LPFG device. This approach has not yet been fully explored, although interesting results have already been obtained with CO_2_ laser interference patterns.

**Figure 3.**
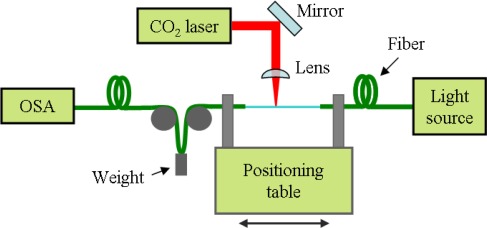
Schematic of the basic point-by-point set-up for laser writing of a LPFG.

**Figure 4.**
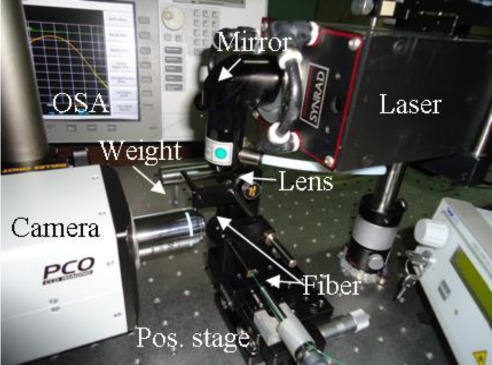
Set-up used to CO_2_ laser writing a LPFG on a Corning SMF-28 fiber.

We have started studying the simple point-by-point laser writing on a common Corning SMF-28 fiber. In Figure 3 and 4 we present the schematic and picture of the implemented set-up.

The fiber is periodically moved along its axial direction with a translation stage and is periodically irradiated by the beam from the laser source (Synrad 48-2). The laser beam is focused on the fiber with a focal diameter at least equal to the diameter of the fiber. Among other possible focusing solutions, it was also used a cylindrical lens thus reducing alignment precision requirements, although increasing the necessary delivered power or increasing the exposure time. In order to induce a constant strain to the fiber, a small weight (usually, a few g) is attached on one of the sides of the fiber [7]. A broad band light source (Thorlabs S5FC1005S) and an optical spectrum analyzer (OSA) allows monitoring the LPFG fabrication, while a fast camera (PCO SensiCAM), perpendicular to the irradiation axis, was used to optically visualize the process. The irradiated zones were analyzed through optical microscopes with amplifications ranging from 5× to 1,000×.

**Figure 5.**
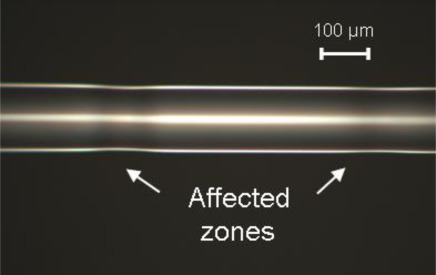
Picture showing two consecutive irradiated areas with almost no change in the cladding thickness. (Spherical lens, exposure time 4 ms, 0.5 W laser power).

**Figure 6.**
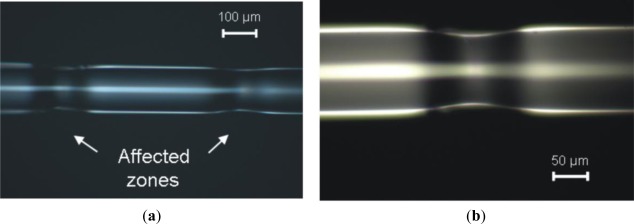
Picture showing (**a**) two consecutive irradiated areas with symmetrical change in the cladding thickness and (**b**) a zoom of one of zones. (Spherical lens, exposure time 400 ms, 1 W laser power).

**Figure 7.**
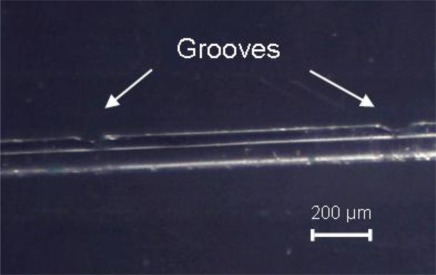
Picture showing two consecutive unsymmetrical changes in the cladding thickness produced by MIR CO_2_ laser irradiation of a Corning SMF-28 fiber. (Cylindrical lens, exposure time 40 ms, 9 W laser power).

Illustrative results are presented in Figures 5–7. The laser used is a 25 W maximum power CW CO_2_ laser (10.6 μm wavelength) electronically modulated, with exposure times in the order of a few hundreds of ms. Light was focused on the fiber by a 50 mm focal length spherical lens or by a 250 mm focal length cylindrical lens. The spot sizes are approximately 0.2 mm and 0.4 mm respectively, measured through the burn spot analysis method [11].

While Figure 5 shows two consecutive refractive index changes with minimum cladding thickness change, Figures 6 and 7 shows the occurrence of symmetric and unsymmetrical changes, respectively. These two latter cases result from a balance between laser power and pulse in order to achieve melting, thus creating tapers or grooves along the fiber (*i.e.*, zones were the cladding diameter is reduced). In these cases, based on what was reported by Wang *et al.* [12], we do not expect a large insertion loss because the groves are generated in the outer cladding and do not influence the light transmitted by the core.

**Figure 8.**
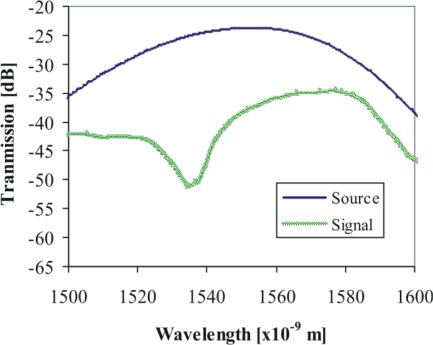
Transmittance spectrum of the broad band source used in the tests and an example of a signal obtained by refractive index modulating the fiber (grating manufactured under the conditions of Figure 6, with a period of about 500 μm and 50 periods).

An example of a signal obtained by symmetrically changing the cladding diameter can be seen in Figure 8. The LPFG was created with a period of about 500 μm; 50 periods were written. These preliminary results are consistent with what should be expected (period and size of the LPFG) although the coupling between the light source and the fiber should be improved in order to reduce the attenuation of the signal and reduce the background loss.

**Figure 9.**
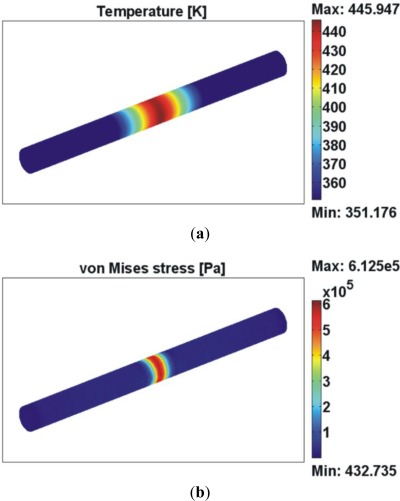
FEM simulation of (**a**) temperature and (**b**) von Mises stress distribution (color scale) for a 1 s irradiation of a 125 μm diameter silica fiber by a 35 mW CO_2_ laser beam. The beam is considered has having a spot size coincident with the fiber’s diameter.

An example of a FEM analysis is presented in Figure 9. This simulation was made with COMSOL multiphysics™ and considered a 35 mW irradiation for 1 s. The fiber was simulated by a cylinder of silica (10^3^ cm^−1^ absorption coefficient at 10.6 μm wavelength); the cylinder has a diameter of 125 μm and a length (just for simulation purposes) of 3 mm. The ambient temperature is considered to be 295 K. The 3D mesh was divided in three volumes in order to allow a tighter mesh size in the central part of the fiber (where the laser beam incidence is simulated) without increasing the computational load. In the central part of the fiber, the mesh was defined by 12,411 tetrahedral elements (0.0439 element volume ratio). The initial condition, at t = 0 s, is that the temperature of the piece is uniform and equal to the ambient temperature (295 K). The analysis includes thermo-mechanical modeling which not only allows studying the temperatures distribution in the silica fiber due to laser irradiation but also the stresses generated within the bulk material. Up to the moment, the existence of the pre-strain and the inner strain of the fiber due to the selective doping solely in the core region were not implemented in the model. This will be made in future developments.

## 4. Laser Micropatterning

Laser micropatterning has been the basis of many of the developments of fiber-based sensors. Optical fiber Fabry-Perot (FP) interferometric sensors and optical fiber surface plasmonic resonance (SPR) sensors are perhaps the fiber-based sensors that can better take advantage of micropatterning optical fibers.

Optical fiber Fabry-Perot (FP) interferometric sensors are the main cavity-based type of fiber-sensors, and have been well demonstrated for various sensing applications in the past [13–15]. The FP cavity can be either intrinsic (e.g., a section of fiber between two dielectric mirrors [16]) or extrinsic (e.g., an air gap between two cleaved fiber end access [17]), although some studies have demonstrated the viability of producing hybrid structured FP interferometers [18]. The reflections at the two end surfaces of the FP cavity create an interference signal which is a function of the length and refractive index of the cavity. Then, changes in environment causes a phase shift in the interference pattern and, as a result, a fiber FP sensor is capable of measuring various parameters including temperature, pressure, strain, acoustics and flow. Their small size, immunity to electromagnetic interference (EMI), and corrosion resistance, makes these FP fiber sensors particularly attractive for applications in harsh environments.

In a more recent development [19,20] microestructured optical fiber (MOF) can be used as a surface plasmon resonance (SPR) sensor. One of the possible architectures, as presented by Hassani *et al*. [20] is to open several holes in the top of a fiber. The fiber core is surrounded by two layers of holes in which the outer (relative to the center) layer consists of metallized holes (for plasmon excitation). These are considerably larger than those in the inner layer, thus simplifying the flow of analyte through them. Air-filled holes in the first layer enable guiding in the higher refractive index fiber core, while at the same time, control the coupling strength between the core mode and the plasmon wave. A small air-filled hole in the fiber core is used to reduce the refractive index of a core-guided mode to facilitate phase matching with the plasmon.

In either FP or SPR cases, laser micropatterning has proven to be an important development or even manufacturing tool. The laser micropatterning has been done mainly by using UV beams [21,22] or femtosecond near-infrared (NIR) laser beams [23,24]. In the first case, single photon interaction is responsible by material removal, while femtosecond pulses promote multiphotonic interactions. In both cases thermal effects are avoided, thus reducing the probability of damaging the fibers. However, such sources are still expensive or deliver low energy. Based on past experience of LOLS with NIR ns beams and their interaction with glasses [25,26], we report here some patterning results using a 1064 nm wavelength beam at nano-second regime [27,28].

Tests were made by irradiating optical fibers with a 7 ns pulse width, 10 Hz repetition rate, Q-switched Nd:YAG laser (BMI, 1,064 nm wavelength) beam. The set-up was similar to the one represented in Figure 4, modified in order to irradiate the top of the fiber. The main goal was to produce cavities on top of Corning’s SMF-28 single mode silica fibers; nevertheless, some tests have also been made to drill apertures in fiber’s side, both in the mentioned fibers (mainly to test the procedures) and on hollow-core type of fibers, which could lead to more sensitive sensors.

**Figure 10.**
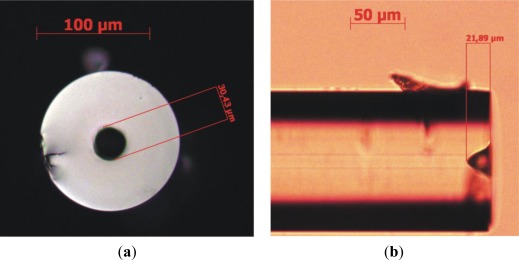
Example of a hole opened on top of a Corning SMF-28 fiber by multiple-pulse irradiation with a focused NIR laser beam. (**a**) Front and (**b**) lateral views taken with an optical microscope. (20 pulses with 3 mJ/pulse).

In Figure 10 we show an example of a hole opened on top of a Corning SMF-28 fiber. In this case, 20 laser pulses were applied, each with about 2 mJ energy corresponding to a fluence of about 8 mJ/cm^2^.

**Figure 11.**
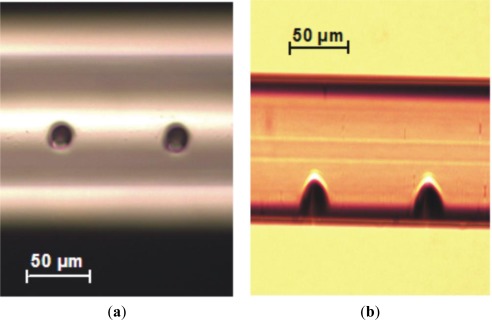
Example of two holes opened on the lateral side of a Corning SMF-28 fiber by multiple-pulse irradiation with a focused NIR laser beam. (**a**) Front and (**b**) lateral views taken with an optical microscope. (20 pulses with 3 mJ/pulse).

Tests demonstrated that the achieved depth has a direct relation with the number of pulses, about 1 μm depth per pulse [27]. It also turned out that neither moving the fiber after each pulse (towards the focusing lens) nor changing the beam size on the focusing lens (but keeping the fluence constant) has major influence on the results. Changing the beam’s energy between 1 mJ and 2.5 mJ was shown to increase the hole diameter on top of the fiber by about 5 μm but not significantly altering its depth (considering the associated measurement errors). Figure 11 shows the results for lateral laser shooting with the same beam parameters.

These results are challenging because these silica-based fibers are mainly transparent to NIR radiation (the absorption coefficient is around 1 dB/km) and therefore the usual explanation based in direct heating by molecular or matrix vibrations induced by the laser beam (as in the previous section) should not hold. This leads to the necessity of a further in-depth analysis of the physical mechanisms involved in the interaction.

One possible alternative to the production of SPR sensors, while maintaining the same physical principle, is to replace the *a posteriori* metallization of the holes by direct formation of metallic nanoparticles simultaneous with the laser micropatterning of the top of the fiber. This requires a metallic ion-doped fiber top. Some successful experiences were already made (in cooperation with the research unit Glass and Ceramic for the Arts—VICARTE) using NIR laser radiation, in the nanosecond pulsed regime to obtain gold and copper nanoparticles in glass substrates [25,28], the first time (to authors knowledge) that these effects were reported in this ns irradiation regime.

## 5. Conclusions

Previous experience in optics and laser processing is being applied to develop new solutions to fiber-based sensors, even with relatively inexpensive Nd-YAG or CO_2_ laser sources, for which cheaper and simpler technology can be found. Intensive engineering activity to support industry has also been instrumental to design, build and test fiber-based sensors in aqueous natural environments and to the development of an encapsulation prototype to be operated in real conditions.

LOLS strategy has become more and more focused in laser micropatterning, use of CO_2_ structured light and generation of metallic particles on fibers to enhance plasmonic effects which boost fiber-sensing sensitivity. Preliminary tests described in this report show the relevance of the metrology to ensure the adequate control of the position of the holes, both on the top or around the fiber and the importance to have adequate simulation models to guide the laboratorial testing. The studies made up to now allowed not only to design and implement promising methodologies but also to identify the challenges to overcome.
